# The African measles and rubella laboratory network: 21 years of functioning in West Africa

**DOI:** 10.11604/pamj.2025.52.126.49605

**Published:** 2025-11-25

**Authors:** Annick Dosseh, Ado Bwaka, Balcha Masresha

**Affiliations:** 1 World Health Organization, Inter-Country Support Team for West Africa, Ouagadougou, Burkina Faso,; 2 World Health Organization, Regional Office for Africa, Brazzaville, Congo

**Keywords:** Measles surveillance, rubella, measles laboratory, laboratory network, serology, Africa

## Abstract

The WHO (World Health Organization) African Region measles/rubella laboratory network was established to provide support to the measles/rubella elimination program. From the official designation of a laboratory to the provision of basic equipment and training, regular laboratory assessment, the network has been functioning for more than 21 years in a standardized manner. We analyzed the performance of the laboratory network in the Western Africa Subregion. The comparison of the period 2004-2013 versus the period 2014-2024 shows an increase in the average number of functioning laboratories, specimens received /tested and onsite accreditation reviews conducted. The overall timeliness in sending out IgM results to EPI/national level remains above the expected target. Quarterly confirmatory testing for serology is implemented by most of the laboratories. More than 92% of the laboratories have obtained a passing score for annual serology Proficiency Test. With the molecular tests taking place in laboratories that have passed the molecular external quality assurance test, measles B3 genotypes were identified in 11 countries of the sub-Region over the past 21 years. As of today, the measles and rubella laboratory network in West Africa continues to operate despite the various challenges faced.

## Brief

The African measles and rubella laboratory network coordinated by the World Health Organization (WHO) of the African region is part of the Global Measles and Rubella Laboratory Network [1]. With 52 laboratories distributed across the WHO African Region (WHO/AFR), the network has been supporting the measles/rubella elimination program since 2002 [2]. The support through the WHO has focused on ensuring standardization of tools and methods (testing algorithm, diagnostic kits, external quality assurance, laboratory procedures, performance monitoring indicators) in the functioning of this network.

In the Western African sub-region, 16 of the 17 countries have at least one designated measles/rubella national laboratory (NL). There is one Regional Reference Laboratory (RRL) located in Côte d'Ivoire with specific functions. Over the past 10 years, an average of 20 NLs were functional as compared to 17 laboratories during the period 2004-2013. This is mainly due to Nigeria, which currently has 8 laboratories included in the network. All the laboratories have at least serology capacity, while selected laboratories, including the RRL have additional capacities (molecular, sequencing, virus isolation). Once a laboratory is officially designated by the national authorities to serve as a NL, laboratory diagnostic & data management trainings are provided by WHO in collaboration with the US Centers for Disease Control and Prevention. On average, one training (onsite country laboratory trainings as well as intercountry workshops) was conducted annually for laboratory staff. Basic serology equipment (including ELISA reader & washer, incubator), supplies (micropipettes, tips, tubes, cryovials), reagents (IgM diagnostic kits) & funds for laboratory operational costs were also provided to the laboratories as a “start up support”.

The serological laboratory diagnostic algorithm requires that upon reception of a blood specimen, each laboratory runs first the measles IgM test and then rubella IgM test on measles IgM-negative specimens. Moreover, measles IgM equivocal specimens are tested twice before releasing the results. The average annual number of blood specimens received in the laboratories in Western Africa Subregion has sharply increased during the past 10 years to 11576 specimens per year, as compared to an average of 513 specimens during the period 2004-2013 (Table 1). Subsequently, the same increase is also observed on the average number of specimens run on measles and rubella IgM. It has been more challenging for the laboratories to meet the expected timeliness in sharing IgM results with the national level (target = within 7 days following the reception of the specimen at the laboratory) during the past 10 years (81%) compared to the period 2004 -2013 (87%) (Table 1). Shortage of diagnostic kits has been frequently observed in some laboratories due to the increased number of specimens received.

**Table 1:** summary of activities and performances of the WHO/AFR measles and rubella laboratory network, West Africa, year 2004-2013 versus year 2014-2024

Indicators	Year 2004 - Year 2013	Year 2014 - Year 2024
Average number of functioning laboratories by year	17 [15-18]	20 [18-23]
Average number of blood specimens received at the laboratory by year	513 [138-1340]	11576 [848-34334]
Average number of blood specimens tested on measles IgM by year	504 [135-1339]	10381 [726-33027]
Average number of blood specimens tested on rubella IgM by year	270 [112-467]	6889 [434-22388]
Timeliness in sending out IgM results to EPI/national level (%)	87	81
Average number of trainings conducted	1 [0-2]	1 [0-2]
Proportion of laboratories with passing score to MR serology PT	97 [80-100]	93 [71-100]
Average number of laboratories that shipped at least one batch of serum specimens to RRL for quarterly confirmatory testing	12 [9-16]	12 [8-19]
Average number of onsite accreditation review conducted	4 [2-6]	7 [0-10]

[ ]= [minimum-maximum]

As part of the external quality assurance (EQA) procedures set up by WHO/AFR, quarterly confirmatory testing for serology (shipment of 10% of sera tested at NL to RRL for retesting) has been implemented by an average of 12 laboratories that have shipped at least one batch of serum by year (Table 1). Measles and rubella (MR) serology Proficiency Test (PT) is a mandatory annual EQA that should be performed by all laboratories. The overall proportion of laboratories with a passing score is generally high although suboptimal performances (71%, 80%) were observed in some years (Table 1). MR molecular EQA (mEQA) is performed by selected laboratories: at the end of 2024, only 3 laboratories (Côte d'Ivoire, Ghana & Algeria) were going through mEQA.

Annual MR laboratory accreditation exercise is another requirement of the network with an onsite review to take place every 3 to 4 years. Since 2004, the average number of onsite reviews for accreditation has increased with an average of 7 reviews conducted by year during the last 10 years (Table 1). At the end of 2024, two-thirds (68%) of the laboratories had provisional accreditation status. Collaboration between the NL and the EPI/surveillance team is a key component of the surveillance system in the countries, particularly when it comes to the investigation of suspected measles/rubella cases and the collection of blood specimens during suspected MR outbreaks. This collection procedure and protocol are outlined in the WHO/AFR measles surveillance guidelines and take into consideration epidemiological linkage. In addition, throat swab specimens are expected to be collected during suspected outbreaks, but this does not take place as required. Over the past 21 years, measles B3 genotype has been identified and submitted at least once on the global MeaNS platform from Algeria, Benin, Burkina Faso, Côte d'Ivoire, Ghana, Guinea, Mauritania, Nigeria, Senegal, Sierra Leone and Togo. D4 and D8 measles genotypes were also identified in Algeria.

The MR laboratory network in West Africa continues to operate and support the African Regional measles and rubella elimination efforts, despite the challenges related to the inadequate funding to support the network, shortage of diagnostic kits due to various reasons. The continued support of national governments and donor agencies will be critical towards sustaining the functionality of the measles and rubella laboratory surveillance network.
